# Supplementary material for: Regulation of NADPH-dependent Nitric Oxide and reactive oxygen species signalling in endothelial and melanoma cells by a photoactive NADPH analogue
Source: Oncotarget. 2014 Sep 25;5(21):10650–64. doi: 10.18632/oncotarget.2525 (PMC4279400; doi:10.18632/oncotarget.2525)
Supplement: Supplementary file 1 [file oncotarget-05-10650-s001.pdf]

Regulation of NADPH-dependent Nitric Oxide and reactive oxygen species signalling in endothelial and melanoma cells by a photoactive NADPH analogue

Florian Rouaud[1], Miguel Romero-Perez[2], Huan Wang[3], Irina Lobysheva[2], Booma Ramassamy[4], Etienne Henry[3], Patrick Tauc[3], Damien Giacchero[5], Jean-Luc Boucher[4], Eric Deprez[3], Stéphane Rocchi[1] and Anny Slama-Schwok[6]

[1] INSERM U1065 team 1, Université de Nice Sophia Antipolis et Centre Méditerranéen de Médecine Moléculaire, Nice, France

[2] Pole of Pharmacology and Therapeutics, FATH5349, IREC, UCL Medical Sector, Brussels, Belgium

[3] Laboratoire de Biologie et Pharmacologie Appliquée (LBPA), ENS-Cachan, CNRS UMR 8113, IDA FR3242, Cachan, France

[4] CNRS UMR 8601, Université Paris Descartes, 45 rue des Saints Pères, Paris, France

[5] Service de Dermatologie, Hôpital Archet II, CHU Nice, France;

[6] Virologie et Immunologie Moléculaires, UR 892, INRA, Jouy en Josas, France

Correspondence to: *Anny Slama-Schwok*, ***email***: *Anny.Schwok@gmail.com*

Stéphane Rocchi, ***email***: *stephane.rocchi@unice.fr*

Keywords: Cellular signaling; Angiogenesis; NADPH analogue; cell proliferation; ROS; melanoma, endothelium

Received: *August 04, 2014* ***Accepted***: *September 24, 2014* ***Published***: *September 25, 2014*

This is an open-access article distributed under the terms of the Creative Commons Attribution License, which permits unrestricted use, distribution, and reproduction in any medium, provided the original author and source are credited.

ABSTRACT

Nitric Oxide (NO) and Reactive oxygen species (ROS) are endogenous regulators of angiogenesis-related events as endothelial cell proliferation and survival, but NO/ROS defect or unbalance contribute to cancers. We recently designed a novel photoactive inhibitor of NO-Synthases (NOS) called NS1, which binds their NADPH site *in vitro*. Here, we show that NS1 inhibited NO formed in aortic rings. NS1-induced NO decrease led to an inhibition of angiogenesis in a model of VEGF-induced endothelial tubes formation. Beside this effect, NS1 reduced ROS levels in endothelial and melanoma A375 cells and in aorta. In metastatic melanoma cells, NS1 first induced a strong decrease of VEGF and blocked melanoma cell cycle at G2/M. NS1 decreased NOX_4 and ROS levels that could lead to a specific proliferation arrest and cell death. In contrast, NS1 did not perturb melanocytes growth.

Altogether, NS1 revealed a possible cross-talk between eNOS- and NOX_4 –associated pathways in melanoma cells via VEGF, Erk and Akt modulation by NS1 that could be targeted to stop proliferation. NS1 thus constitutes a promising tool that modulates NO and redox stresses by targeting and directly inhibiting eNOS and, at least indirectly, NADPH oxidase(s), with great potential to control angiogenesis.

INTRODUCTION

A critical pathogenic factor in the development of endothelial dysfunction is redox stress involving generation of reactive oxygen and nitrogen species, RNOS. Identifying sources of excessive or unbalanced RNOS levels and developing alternative strategies to regulate their respective levels should help in designing novel rational therapies. Previous studies identified NADPH oxidases of the NOX family, the respiratory chain in mitochondria and uncoupled endothelial NO-synthase (eNOS) as major sources of reactive oxygen species (ROS) in endothelial dysfunction [1-3]. Superoxide ions and H_2O_2 share with nitric oxide (NO) a dual role in cytotoxicity and signalling at high and low concentrations, respectively [4]. The signalling pathways regulated by NO formed by eNOS are linked with activation of soluble guanylate cyclase and vasodilation [5, 6]. NO is also involved in pathways regulating cell survival in response to lipid or oncogenes (KRas) (PI3K/ Akt/ eNOS

pathway), to low oxygen tension (HIF/VEGF/ Akt/eNOS pathway), to metabolic stress (AMPK/eNOS pathway). eNOS is regulated at the transcription level by NF-κB, p53 and by post-translational modifications. Through a cross-talk between them, NO and ROS participate in redox homeostasis in healthy cells, a balance that is often deregulated by pathogens and in pathophysiological situations. In some cancers, NO acts as an endothelial growth factor that mediates tumor growth and metastasis [7, 8]. Inhibition of ROS formed by NADPH oxidases (NOX) and/ or by eNOS uncoupling is highly requested for pharmacological treatments of oxidative stress associated with cardiovascular diseases and cancers [9]. Most current NOS inhibitors designed based on X-ray structures target the heme site [10]. However, these inhibitors cannot prevent ROS formed at the reductase domain level. The design of NOS inhibitor targeting the reductase domain could lead to "two in one" effects by inhibition of both NO formation and undesirable ROS production from NOS uncoupling.

A rational way to identify sources of redox stress would be to make use of compounds modulating NADPH levels, which requires selectivity toward specific NADPH-dependent enzymes without strong interference with normal cellular processes. Recently, we designed a novel photoactive probe, called nanoshutter (NS1) that efficiently bound to constitutive NOS (eNOS and nNOS) by recognition of their NADPH binding site [11] in a similar manner than our previously reported dienic nanotrigger [12-15]. NS1 reversibly inhibited NO formed by recombinant NOS by competing with NADPH binding [11]. Moreover, while NS1 was not fluorescent in aqueous solutions, a specific enhancement of NS1 fluorescence upon NS1 binding to constitutive NOS was observed upon two-photon excitation. These fluorescence properties allowed NS1 imaging of eNOS in living HUVEC cells at the cell membrane and at the Golgi levels with minimal contribution of flavin autofluorescence [11].

In the present manuscript, we showed that NS1 inhibited NO formation and modulated ROS levels in endothelial cells, isolated aorta and melanoma cells. We tested how NS1 affected angiogenesis and metastatic melanoma cells growth, two processes dependent upon NO and ROS. NS1 probed the relationships between NOS and NOX in endothelial and melanoma cells resulting in anti-angiogenic effects, targeted metastatic melanoma cell growth arrest while being non-toxic to normal melanocytes. NS1 combined interesting pharmacological and imaging properties, with great interest as a probe of key redox mediators and for future anti-angiogenic therapies.

RESULTS

NS1 inhibits the formation of NO produced in isolated mice aortic rings

We have previously shown that NS1 targeted purified NOS and inhibited NO production competitively relative to NADPH. Here, we addressed the question of NO inhibition by NS1 in a more physiological context. NO production was assessed by EPR spin trapping as formation of the paramagnetic [Fe(DETC)$_2$NO] complex in isolated mice aortic rings stimulated with ionomycin in the presence of Fe(DETC)$_2$ [16]. Addition of 1mM L-NAME, a NOS inhibitor, fully abolished the formation of NO by stimulated aorta as expected. NS1 decreased formation of NO by aortic rings in a monotonic manner (Figure 1 A, B). A concentration of NS1 inhibiting NO formation by 50%, IC_{50} value of 40 ± 15 µM was measured and corresponded to IC_{50} previously determined for NO inhibition from recombinant eNOS or nNOS, IC_{50} = 30 ± 10 µM [11]. The *in vitro* IC_{50} yielded an inhibitory equilibrium constant Ki being Ki= 4 ± 1 µM. IC_{50} value for heme-based inhibitors as L-nitro-arginine are usually in the micromolar range while IC_{50} for L-NAME being 70 µM [17, 18].

NS1 inhibits VEGF-dependent angiogenesis of HUVECs

To assess the effects of NS1 on angiogenesis, a physiological eNOS-dependent process, an assay of endothelial network formation (plating of HUVECs on matrigel) induced by VEGF was used. The formation of cord capillary structures in the absence or presence of 1% serum as previously reported [19, 20] was monitored as a function of time (Figure 1C and supplementary Figure 1A, respectively). A significant increase in capillary structures formation by HUVECs was already observed after 12 h. The addition of increasing concentrations of NS1 to the cells led to a reduction of their ability to form capillary structures (Figure 1D-E). 100µM NS1 strongly reduced the rate and the amount of tube formation and the number of crossings between them compared to control experiments; the significant differences between groups demonstrated the anti-angiogenic effect of NS1 on these endothelial cells.

NS1 inhibits H_2O_2 and superoxide formation by NOS under uncoupling conditions

NS1 was expected blocking the electron flow in NOS. Therefore, NS1 should avoid ROS formed under uncoupling conditions. NOS can generate ROS from O_2

reduction by flavins of the reductase domain and from the heme site by dissociation of the Fe^{II}-heme-O_2 complex in the absence of substrate and/or cofactor H_4B to form superoxide and regenerate Fe^{III}-heme (uncoupling) [21]. We tested the effects of NS1 on the levels of hydrogen peroxide and superoxide ions formed by uncoupled nNOS. H_2O_2 was measured in a colorimetric assay and $O_2^{\cdot-}$ monitored by EPR spectroscopy using spin-trapping experiments in the presence of the cyclic nitrone DEPMPO. In the absence of substrate and with low amounts of H_4B, H_2O_2 formation by nNOS was 145 ± 22 nmol.min^{-1}.mg prot^{-1}, which was inhibited by NS1 with an IC_{50} value of 75 ± 12 µM (not shown). Accordingly, uncoupled nNOS catalysis led to the gradual appearance of the characteristic 8-lines features on the EPR spectra corresponding to the nitroxide DEPMPO-OOH spin-adduct (Figures 2A and 2B). The rate of formation of the spin-adduct was normalized to 100 in the absence of L-arginine and H_4B. As expected, this rate was reduced by the addition of 100 µM arginine and 10 µM H_4B and also inhibited by the addition of NS1 with an IC_{50} = 105 ± 15 mM without formation of other detectable paramagnetic species (Figure 2B, C). The results supported that NS1 inhibited electron leakage in nNOS as expected from NS1 design that targets the reductase domain and blocks the overall electron flow to the heme in nNOS by acting at the initial step of electron injection to FAD. We then investigated whether NS1 may affect ROS levels in endothelial cells, and in isolated aorta.

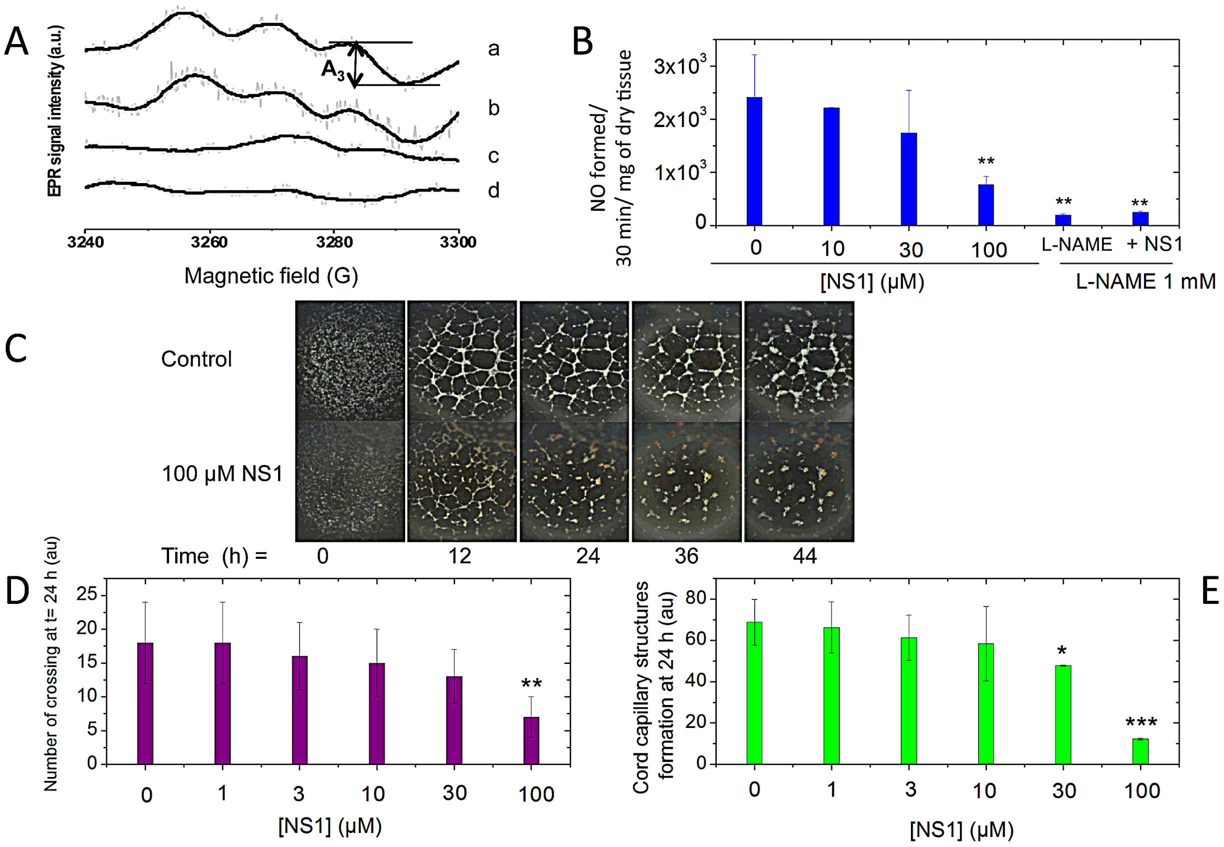

Figure 1: A-B: NS1 inhibited NO formed in isolated aorta rings. Aorta rings isolated from C57BL/6J male mice, 15 weeks old were incubated for 2 h with NS1 ([NS1] = 0, 10, 30, 100 µM) and then stimulated with 2 µM ionomycin. A-Formation of the $Fe(DETC)_2NO$ complex was detected by EPR spectroscopy following 30 min incubation in the presence of $Fe(DETC)_2$ complex as described under Experimental procedures. L-NAME was used as a control. Typical EPR spectra of $Fe(DETC)_2NO$ complex formed after 30 min incubation of aortic rings with $Fe(DETC)_2$ spin trap and 2 µM ionomycin (a), or pretreated with 30 µM (b) or 100 µM NS1 (c) or in presence of 1 mM L-NAME (d). The intensity of third hyperfine component (A_3) was used for the EPR signal quantitation. B: Results are expressed as NO formed (arbitrary units) / 30 minutes / mg of dry tissue and are means ± SD from 3-6 experiments. **C-E: Effects of NS1 on a 3D-model of angiogenesis using VEGF-stimulated HUVECs grown on matrigel**. C: Comparison of the kinetics on endothelial tube sprouting without and with 100 µM NS1 in the absence of serum (see also Supplementary figure 1 for data in the presence of 1% serum); D: Quantification of the experiments (n=3): length of the tubes formed in 24h in serum free in the presence of 0, 1, 3, 10, 30 and 100 µM NS1. Results are expressed as endothelial network percentage ± SEM versus control condition and *n* reflects the number of experiments. E: number of crossings formed in 24h in the same experiments as in D. Significant differences between groups were calculated by ANOVA followed by a Bonferroni test.

Effects of NS1 on ROS formation in HUVECs detected by a fluorescent probe

The effect of NS1 on ROS formation in HUVECs cells was addressed by performing flow cytometer experiments using the CellROX® Deep Red oxidative stress probe (Figure 3A-B). ROS formation is shown by an enhancement of the probe fluorescence (absorption/emission maxima at ~644/665 nm) as observed using tert-butyl hydroperoxide (TBHP) as a positive control for ROS formation (Figure 3A, lower panel). Fluorescence signals of NS1 and CellROX® Deep Red were measured by using FL-1 and FL-4 channels, respectively. To minimize differences in basal cellular ROS among different experiments, the fluorescence signal in the presence of NS1 was normalized by the signal monitored in the same cells without NS1. This normalization gave a fluorescence enhancement factor (FEF), which accounts for ROS formation as a function of NS1 concentration (Figure. 3B). Interestingly, ROS detection in HUVECs presented a decreasing phase at NS1 concentrations above 5 µM (Figure 3B) characterized by FEF values below 1, indicating that NS1 inhibited the basal production of ROS in HUVECs by roughly 50%.

Effect of NS1 on the formation of superoxide ions by mice aortic rings detected by EPR

To test the effect of NS1 on ROS species formed in aorta, the CAT1-H EPR spin probe was used for

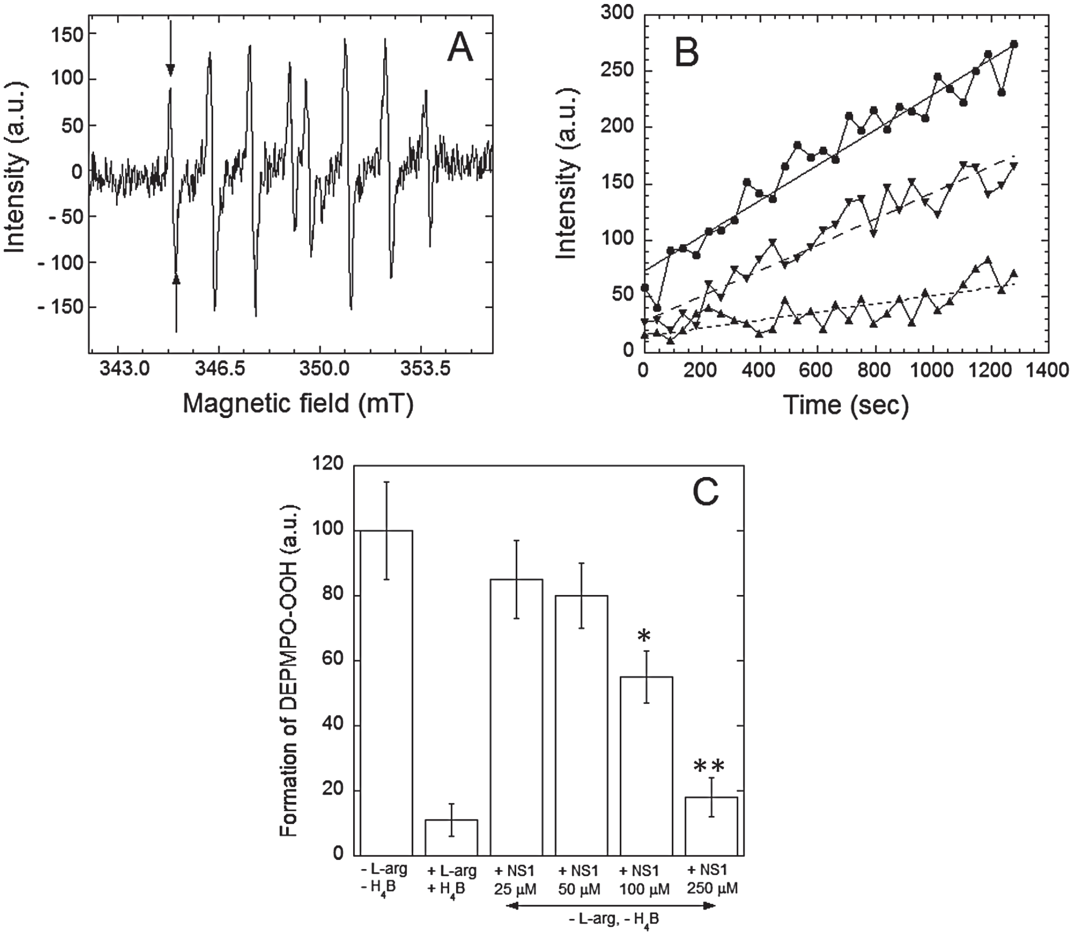

Figure 2: Effects of NS1 on the rates of formation of superoxide anion by nNOS. Superoxide anion was trapped by DEPMPO and the DEPMPO-OOH spin-adduct was detected by EPR spectroscopy. A: Spectra of DEPMPO-OOH spin-adduct formed upon production of $O_2^{\cdot-}$ by nNOS *in vitro* for 15 min under conditions described in Experimental procedures. Horizontal axis: magnetic field intensity; vertical axis: intensities of the EPR signal (arbitrary units). B: Rates of formation of the DEPMPO-OOH spin adduct by uncoupled nNOS in buffer alone (●) or in the presence of 100 (▼) and 250 µM (▲) NS1. Intensities of the first line of the DEPMPO-OOH spin-adduct (arrows on Fig. 2A) are reported as a function of time. Linear fitting of the data points are shown as (–), (- -) and (···), and the respective slopes are 0.156, 0.068 and 0.036. Data from a representative experiment. C: Spin adduct levels formed after 15 min incubation of nNOS under various experimental conditions. Data are normalized to the level of spin adduct formed in the absence of substrate L-arginine and cofactor H_4B. Data are means from 3-4 experiments.

measurement of superoxide ions. This probe cannot easily cross cellular membrane and is oxidized by ROS to paramagnetic CAT1● radical detected by EPR spectroscopy. The kinetic of radical formation was detected by the EPR as indicated in Experimental procedures and typical EPR spectrum of CAT1● radical formed after oxidation by $O_2^{\cdot -}$ is presented in an insert (Supplementary Figure 2). Pre-incubation of mice aortic rings for 30 min on ice with 100 U/mL SOD decreased EPR signals by 80% (Figure 3C). This large signal decrease in the presence of SOD identified superoxide ions, species sensitive to SOD as the predominant species involved in oxidation of CAT1-H in unstimulated aortic rings. Addition of L-NAME (1mM) had no significant effect on the signal (data not shown), consistent with eNOS uncoupling (at the level of the heme domain) not being a major source of superoxide in this model. Isolated aortic rings incubated with 10 µM NS1 inhibited the formation of CAT1● radicals by 60 ± 19 %, a similar level than that observed with SOD within experimental error. The EPR data supported NS1 inhibition of superoxide ions generated by membrane-bound enzymes in aortic rings, in good agreement with ROS inhibition observed with the fluorescent probe in HUVECS at 10 µM NS1 (Figure 3B).

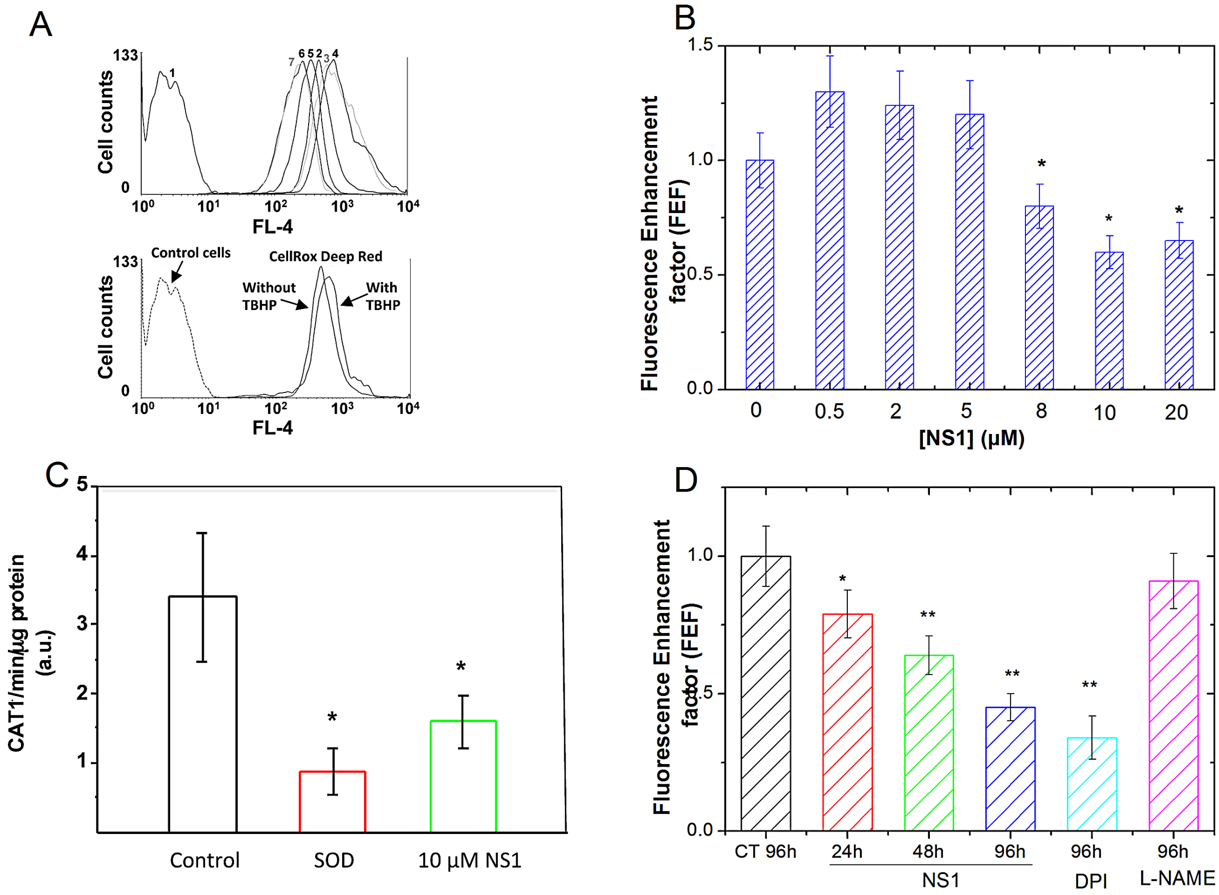

Figure 3: NS1 modulation of ROS formation in HUVECs, aorta and melanoma A375 cells. HUVEC cells were pre-treated with increasing concentration of NS1 for 30 min and further treated with 2 µM CellROX® Deep Red Reagent for 30 min before flow cytometry analysis for intracellular level of ROS measurements. A: Upper panel: One example of cell population distribution of CellROX® Deep Red fluorescence intensity (as measured in FL-4 channel). Curve 1: control cells (in the absence of CellROX® Deep Red and NS1); curve 2: cells + CellROX® Deep Red alone; curves 3-7 (left): HUVEC cells + CellROX® Deep Red in the presence of increasing NS1 concentrations (0.5, 1, 8, 10 or 20 µM, respectively). The oxidative stress inducer TBHP was used at a concentration of 400 µM as a positive control (lower panel). B: Fluorescence enhancement factor of the CellROX® Deep Red Reagent as a function of NS1 concentration. The fluorescence enhancement factor (FEF) corresponds to the ratio between the MFI (Mean Fluorescence Intensity) values obtained in the presence and in the absence of NS1, respectively, as measured in FL-4 channel. The reported ratios correspond to averages of at least four independent measurements. C: Effect of NS1 on the formation of basal ROS by isolated aortic rings in the presence of vehicle alone (control) or after addition of 10 µM NS1. The hydroxylamine probe CAT-1H was oxidized to nitroxide CAT1● radical that was detected by EPR as indicated in Experimental procedures. The basal level corresponded to the oxidation of the probe in the absence of aortic rings (with or without NS1). As expected, the signal was inhibited by SOD. Data are taken from 3-4 representative experiments. D: Kinetics of ROS levels decrease induced by NS1 (30 µM) or DPI (100µM) but not by L-NAME (100 µM) in melanoma cells; the ROS levels are expressed as Fluorescence enhancement factor as in B determined by the CellRox reagent.

Effect of NS1 on ROS produced by A375 melanoma cells

Melanoma cells are characterized by altered redox status, in particular higher ROS levels than required for normal cell signalling [22]. We thus tested whether NS1 may affect ROS levels in metastatic melanoma A375 cells. As in HUVECs, the CellRox reagent was used for quantification of ROS levels changes in the presence of NS1 (30µM), DPI (100µM), or of L-NAME (100µM) (Figure 3D). NS1 induced a time-dependent decrease of ROS, 60% inhibition of ROS levels were observed at 96h, in agreement with a similar decrease observed on HUVECs (Figure 3B). ROS levels were unchanged by L-NAME, suggesting that ROS were not mainly produced by NOS uncoupling at the level of the heme domain. As expected, DPI, an inhibitor of flavoenzymes (as NOX's or NOS reductase) decreased ROS levels. In endothelial cells, NOX_4-derived H_2O_2 promoted proliferation and is activated by ischemia [23, 24]. We then tested how NS1 affected angiogenesis and melanoma proliferation.

Effects of NS1 on A375 melanoma cells angiogenesis and proliferation

Melanoma is characterized by its high propensity to metastasize and its proliferation has been linked to an abnormal metabolism, altered redox signalling pathways in which NOX_4, uncoupled eNOS and sustained

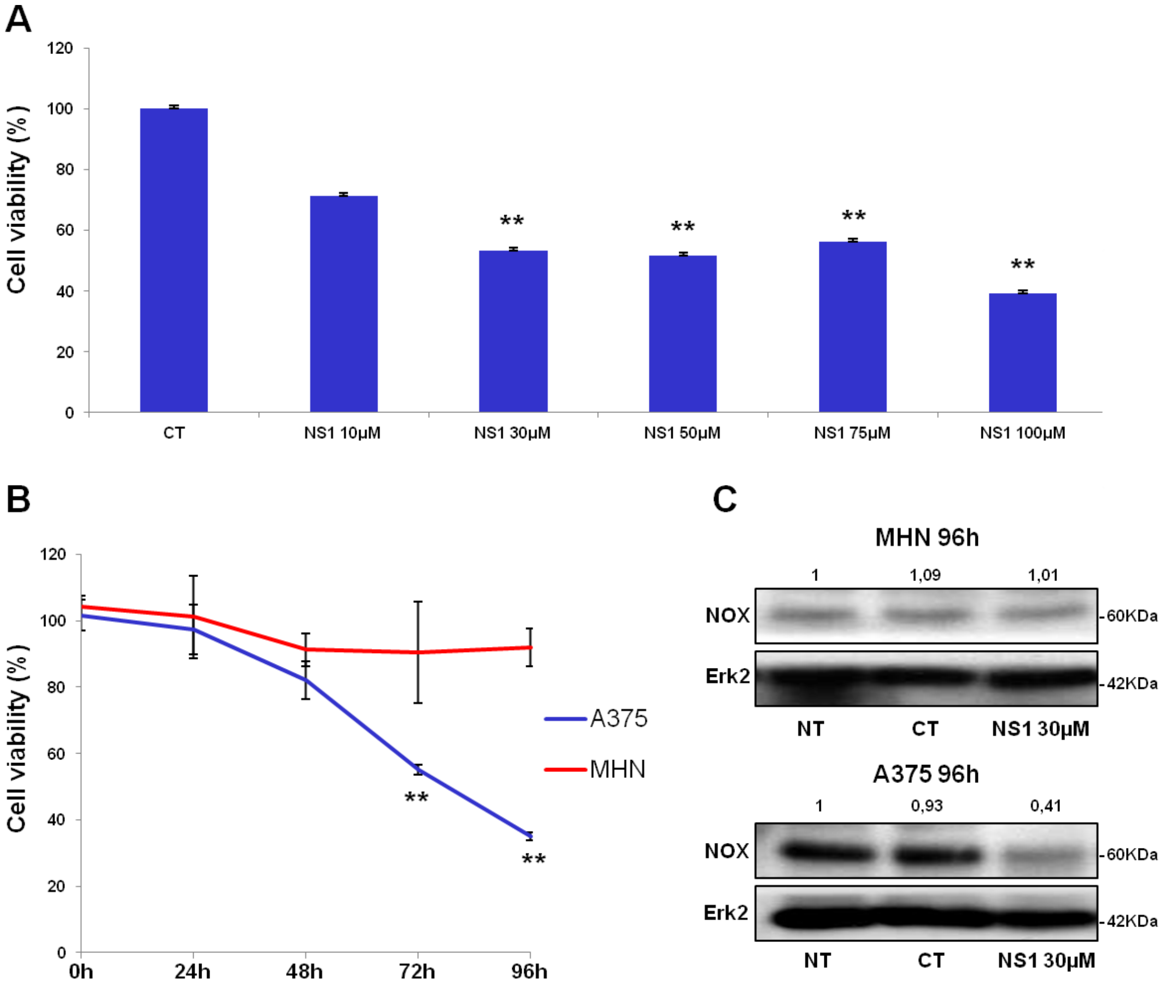

Figure 4: Cell viability and NOX_4 levels with / without NS1: comparison of melanoma cells and melanocytes. A- Effect of increasing concentrations of NS1 on the number of melanoma A375 cells in the presence: melanoma A375 cells were treated with indicated concentrations of NS1. After 96 h incubation, viable cells were counted using trypan blue dye exclusion method. CT: control buffer, NS1: cells treated with the indicated concentration of NS1. B- Kinetics of primary human melanocytes (NHM) and A375 melanoma proliferation treated with 30 µM NS1. Melanoma A375 cell lines or NHM were treated with indicated concentrations of NS1. After 24, 48, 72 and 96 h, viable cells were counted using trypan blue dye exclusion method. For each experiment, cell number is expressed in percent of control (100%). Data are mean ± S.D. of three independent experiments performed in triplicate. C- Western blots revealed the presence of low levels of NOX_4 in NHM cells in the three conditions: untreated (NT), control buffer (CT), and treated with NS1 in the same buffer; in contrast, A375 presented an elevated level of NOX_4 in the untreated and control conditions which strongly decreased in NS1-treated cells. Levels of Erk2 were used as internal control.

iNOS expression were involved [25-27]. Because these enzymes associated with redox stress could be targeted by NS1, we tested whether the number of metastatic melanoma A375 cells decreased as a function of NS1 dose. Normal human melanocytes (NHM) were used as a control. Supplementary Fig. 3A shows that increasing concentrations of NS1 had minimal effect on these healthy NHM cells after 24 and 48 hours. A low toxicity of NS1 in HUVECs was also deduced from minimal effect on MTT tests (Supplementary Fig. 3B) and the lack of morphology change during cell growth recorded by video-microscopy for 24 h (data not shown). These data support the specific effects of NS1 activity on NO and ROS levels in HUVEC and melanocytes.

In contrast, NS1 reduced the proliferation of melanoma cells in a time-and dose-dependent manner (Figure 4). The number of A375 cells decreased upon increasing NS1 concentration (Figure 4A). As shown in Figure 4B, NS1 (30µM) specifically reduced the number of melanoma cells without affecting the growth of melanocytes. We investigated the effect of NS1 on NO derived species (NO_x) levels determined by the Griess reagent. However, NOx were below detection limit (~2µM) in both melanocytes and A375 cells supernatants treated or not with 30 µM NS1 during 96 hours. To investigate whether NS1 may selectively affect NOX_4 levels in melanoma cells compared to melanocytes, western blots were performed in both cell types in conditions in which A375 proliferation was strongly decreased by NS1 (96 hours). Figure 4C shows that NS1 strongly reduced NOX_4 levels in A375 cells and not in melanocytes. NOX_4 overexpression in melanoma cells could be linked with angiogenesis [22, 24]; thus, NS1-induced inhibition of NO and decrease of ROS formed by NOX (Figures 1 and 3) may modify VEGF levels. Figure 5A shows that already after 24h, NS1 (30µM) strongly reduced VEGF levels even before significant changes of cell growth took place in A375 cells compared to melanocytes. After 24h and 48h, treatment of the A375 cells with L-NAME did not affect VEGF or NOX_4 expression After 96 h, L-NAME only decreased VEGF levels without modifying NOX_4 levels. In contrast, VEGF decreased after 48h treatment of the A375 cells with DPI (100µM), compared to a VEGF decrease already observed after 24h treatment with NS1 (30µM). At later times (96h), DPI inhibited VEGF and largely decreased NOX_4 levels, as observed in the presence of 30 µM NS1 (Figure 5A). We thus investigated if NS1-induced changes of ROS and NOX_4 levels were related to modifications of cell cycle as a function of time. An increase of the fraction of cells in G2/M stage and a decrease of cells in the G0/G1 stage were observed after incubation with 30µM NS1 after 48h (Figure 5B). Entry in the cell cycle is initiated by multiple mitogenic stimuli, including Erk and PI3K/ Akt associated with ROS signalling [28, 29]. Accordingly, along with a decrease of VEGF levels, a decrease of phospho-Erk, p21, p27 and associated CDK6 were already observed after 24h (Figures 5A-C). In agreement with a cell cycle pause in G2/M, decrease in ROS levels and phosphorylated retinoblastoma Rb were observed at 48h (Figures 3D, 5C and supplementary Figure. 4). At later stages, NS1 affected both NOX_4 activity and expression, decreased phospho-Akt and increased Akt levels, resulting in an additional increase of cells at subG1 stage, clearly indicated by the decrease of p53, of pro-caspase 3, increase in cleaved PARP (Figure 5C).

DISCUSSION

NS1 is a new prototype of a reversible inhibitor of constitutive NOS targeting their reductase domain [11]. NS1 was designed by molecular modelling, by replacing the imbedded NADP cofactor in nNOS reductase domain [11, 30]. NS1 shares with NADPH the nucleotide moiety that allows proper targeting to the NADPH site; thus, NS1 competes with NADPH binding [11, 13, 15]. In this work, we tested the potential pharmacological applications of NS1 to inhibit angiogenesis and cell growth in physiological and pathophysiological cell models in the endothelium and in melanoma cells, respectively.

NS1 targeted eNOS in the endothelium, inhibited NO-mediated angiogenesis, inhibited uncoupling and decreased ROS levels

NS1 inhibited NO formed by eNOS expressed in isolated aortic rings (Figure 1A-B). The IC_{50} values for NO inhibition corresponded to IC_{50} previously determined *in vitro*, IC_{50} = 30 ± 10 µM [11], a value that should be improved for *in vivo* applications. Improving efficacy and specificity of NS1 by designing isoform-specific inhibitors is ongoing in our laboratories. Accordingly, NS1 modulated endothelial sprouting of HUVEC cells in a 3-D model of angiogenesis, a NO-dependent effect [31]. As expected from VEGF-mediated signalling combined with NS1 inhibition of eNOS, NS1 exhibited anti-angiogenic effects in a dose-dependent manner (Figure 1C-E). These data show that NS1 inhibited angiogenesis, an important NO-dependent process in endothelial cells. These results suggested perspectives at limiting or preventing deleterious effects of NO produced by iNOS or eNOS in metastasis [7, 32].

Decrease of superoxide ions induced by NS1 in aorta while no inhibition by L-NAME was observed (Figure 3C and data not shown) is suggestive that NS1 mainly inhibited active NOX(s) at the cell membrane [23] and that eNOS uncoupling was not a major effect in isolated aorta. Uncoupling of recombinant nNOS was inhibited by NS1, as anticipated from NS1 design as a consequence of NS1 blocking the electron flow in NOS (Figure 2). This constitutes an intrinsic advantage of NS1

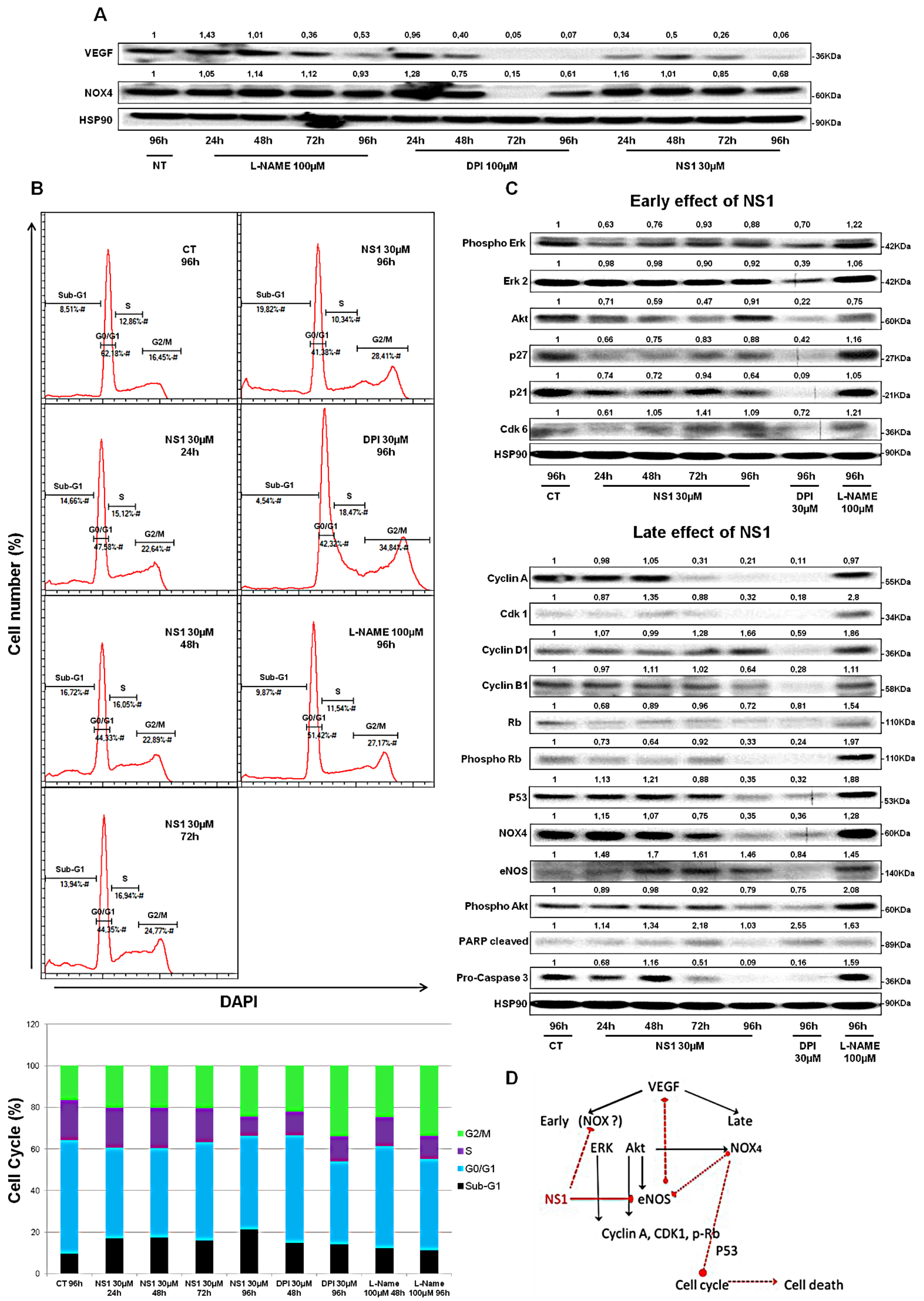

Figure 5: Kinetics of NS1 (30µM) –induced changes on melanoma cells at 24, 48, 72 and 96h: A- angiogenesis as probed by VEGF and NOX_4; B- Changes in cell cycle induced by NS1 or DPI 30µM or L-NAME 100 µM, the upper panel represents one example of cell population determined by FACS analysis, quantified in the bottom panel as an histogram; C- Kinetics of NS1- induced modifications in signalling pathways and cell cycle- associated factors at two timescales defined as early (24 and 48h) and late effects (72, 96h) compared to DPI and L-NAME D- Summary scheme: NS1 affected the Erk then the Akt pathways through direct and/or indirect effects on constitutive eNOS and likely NOX(s), leading to early cell cycle arrest, then late cell death associated with down regulation of p53 and NOX_4. Full red line represents NS1 direct inhibition of eNOS, dashed lines represent suggested NS1(direct or indirect) inhibition of NOX, suggested cross-talk between eNOS and NOX_4 and suggested NOX_4-mediated cell cycle arrest and death.

over other NOS inhibitors directed against the oxygenase domain, unable to avoid uncoupling at the level of the reductase domain. Inhibition of ROS by NS1 (Figure 3B, C) combined with the lack of metabolic activity modification (Supplementary Figure 1) would not suggest a major effect of NS1 (10 µM) on mitochondrial enzymes. Consistent with this effect of NS1 on membrane-bound enzymes, imaging studies showed NS1 localization at the membrane, the golgi apparatus and sometimes in early endosomes in HUVECs [11]. We emphasize that the ROS levels induced by 10-20 µM NS1 decreased by a factor of ~2 with respect to basal level in HUVECS cells and in aortic rings (Figure 3) that likely took place within the physiological range in endothelial cells as confirmed by the lack of viability loss (Supplementary Figure 1).

NS1 specific inhibition of melanoma growth with a lack of toxicity toward melanocytes; Feedback regulation(s) of VEGF-dependent NOX and cross-talk between NOX and eNOS?

Melanocytes are sensitive to ROS imbalance [22]. Oxidative stress contributes to angiogenesis and metastasis involved in the development of tumors. In melanoma, several signalling pathways promoted high ROS levels, therefore melanoma cells established efficient anti-oxydant mechanisms to increase proliferation and avoid cell death [33]. In melanoma A375 cells, NOX_4 was highly expressed compared to low levels in melanocytes (Figure 4C), as reported [27]. NOX_4 is linked to proliferation: in the endothelium, it promotes angiogenesis through eNOS activation [24] and in melanoma, NOX_4 converted radial to vertical growth by overexpression of Akt [34]. In addition to NOX_4, uncoupled eNOS and overexpressed NOX_1 also produced ROS in melanoma [25, 26], linked to Epithelial–Mesenchymal Transition [35].

The growth of normal (primary) human melanocytes was also unaffected by up to 200 µM NS1 (supplementary Figure 1 and Figure 4B) and in these cells, the low NOX_4 levels were not modified by the addition of NS1. NS1 decreased the growth of A375 melanoma cells in a time and dose-dependent manner that we arbitrarily divided into two time windows, early: 24 - 48h and late: 72 - 96 h (Figures 4 B and 5). At early times, treatment of the melanoma cells with 30 µM NS1 induced a decrease of VEGF levels and phospho-Erk (Figure 5A,C). This decrease suggested that NS1, through early direct inhibition of membrane-bound e/nNOS and /or NOX, inhibited the Erk pathway (Figure 5D). In these conditions, L-NAME did not reduce VEGF and phospho-Erk while DPI reduced both. The effects of NS1 on VEGF and phospho-Erk compared with those induced by DPI and L-NAME, taken together with ROS decrease, rather suggested the involvement of NOX in NS1-mediated effects at early times (Figure 3, [36-38]). The increase of eNOS levels at 48h hinted at a regulation in the Akt pathway to "adapt" to ROS decrease (Figures 3D and 5C). Enhanced eNOS levels were observed in NOX-knocked-down coronary vessel [39]. Additionally, an indirect NS1-induced decrease of NOX activity at early times as result of inhibition downstream to Akt cannot be excluded [40]. Whatever the exact mechanism(s) involved, NS1 induced a decrease of VEGF, ROS levels, activated Erk and a cell cycle arrest at G2/M after 48h (Figures 3D, 5C and supplementary Figure 4) [22, 41].

At late times, NS1-induced decrease of ROS levels at the membrane may affect the cytosolic ROS pool [42] since NOX_4 also localized at the ER and the nucleus. ROS generated by NOX_4, was partly depleted by NS1, which likely led to apoptosis in melanoma cells as observed in pancreatic cancer cells [40]. The strong decrease of NOX_4 and phospho-Akt levels at 96 h was concomitant with an increase of Akt, consistent with a mutual regulation of NOX_4 and Akt [43]. In agreement with previous data [41, 44], NOX_4 inhibition paralleled with down-regulation of p53, increase of cyclin D1 and associated Cdk6. Additionally, Akt inhibition and associated strong decrease of cyclin A (Figure 5B, [45]) could be linked with translational control via PERK (-EIF2α) [46] with an observed increase of PERK levels (data not shown) and an increased susceptibility of tumor cells to cell death. Thus, NS1 decrease of NOX_4 expression suggested several ways of (retro)control on NOX_4 activity taking place as revealed by NS1. Feedback loops on NOX activation have been suggested to regulate growth and differentiation of progenitor cells [47] and invoked in propagating vascular damage to smooth muscle cells [48]. While NS1 significantly decreased ROS levels as observed in HUVECs (Figure 3), the sensitivity of melanoma cells to redox changes led to NS1-induced cell death in A375 cells while HUVECs remained viable.

Toward potential targeted therapeutics for melanoma

Novel strategies against melanoma aiming at exacerbation of ROS levels to induce DNA damage and apoptosis have been proposed in combination with inhibition of anti-oxidant enzymes (SOD, catalase and redox transcription factors) [33]. Interestingly, opposite strategies also led inhibiting cancer growth in vivo through ROS (and NOX) inhibition [27, 49]. Currently, a few efficient NOX inhibitors are available that prevented NOX subunit assemblies but isoform-specific NOX inhibitors are still missing [9, 23, 50]. Inhibition of nNOS by isoform-selective compounds targeting the heme site were the first compounds targeting NO that led to inhibition of melanoma cells growth but with some cellular toxicity [51, 52]. Here we hypothesize that, by regulating more than one signalling mediators as NO and ROS through VEGF, Akt

and NOX_4, small targeted-molecules like NS1 may alter redox fluxes between the tumor and its micro-environment and possibly leading to "normalization" of tumor blood vessels [38, 53] and reduction of angiogenesis/ metastasis. NS1 decreased specifically NOX_4 levels in melanoma and not in healthy melanocytes; detailed mechanistic studies are ongoing [54]. The lack of detection of high NO levels by the Griess reagent suggested a minimal involvement of iNOS in NS1 effect [29]. The molecular-targeted mode of inhibition is unique to NS1 design mainly targeting NADPH sites of constitutive NOS and possibly NOX [11-14]. In conclusion, NS1 combined the ability of cellular imaging eNOS in endothelial cells and pharmacological effects allowing a specific decrease of redox stress, in particular in metastatic melanoma that led to stop cell growth. Further in vivo studies are required for testing the potential therapeutic effects of NS1 or additional NADPH derivatives with improved specificity.

Experimental

Reagents: NS1 was synthesized as previously described ([11],[55]). Catalase, calmodulin (CaM), diethyldithiocarbamate sodium salt (DETC-Na), KCl, $Fe(SO_4)_2$, $Na_2S_2O_4$, diethylenetriaminepentaacetic acid (DTPA), Hepes, phorbol 12-myristate 13-acetate (PMA), 3-[4,5-dimethylthiazol-2-yl]-2,5-diphenyl tetrazolium bromide (MTT), L-N^G-nitroarginine methyl ester (L-NAME) were purchased from Sigma-Aldrich (Saint-Quentin Fallavier, France). 5-diethoxyhosphoryl-5-methyl-pyrroline *N*-oxide (DEPMPO) was from Radical Vision (Marseille, France). 1-Hydroxy-2,2,6,6-tetramethylpiperidin-4-yl-trimethylammonium chloride (CAT1-H) was from Enzo Life Sciences Inc. (Farmingdale, NY, USA). DMEM culture media and endotoxin-low fetal calf serum (FCS) were from Gibco-InVitrogen. CellROX® Deep Red Reagent was purchased from Invitrogen. NOX_4 antibody was purchased from Abcam, VEGF antibody from Neomarkers, antibodies against Rb, p53 and Erk and Hsp90 were obtained from Santa Cruz, Cell signaling Technology (CST) provided antibodies against Akt, phospho-Akt (Ser418), p21, p27 kip1, CdK6, cyclin A, cyclin D1, PARP cleaved and BD biosciences anti-eNOS and anti-Pro-Caspase 3; the Griess reagent kit was obtained from Promega. Recombinant rat brain nNOS was expressed and purified as described [56].

Experiments with recombinant nNOS

Hydrogen peroxide released by nNOS was determined by measuring the oxidation of ferrous thiocyanate to ferric thiocyanate as previously described [57]. The reaction mixture contained NADPH (200 µM), $CaCl_2$ (1.0 mM), and CaM (10 µg/mL) in 50 mM Hepes buffer pH 7.4. The incubation was initiated by the addition of aliquots of nNOS (40-100 nM) and stopped after 10 min at 37°C by the addition of 6M HCl; 10 µL of 0.5 M NH_4SCN and 50 µL of 50 mM $Fe(SO_4)$ (freshly prepared) were added to the mixture, the absorbance at 492 nm of the ferric thiocyanate complex was read on a microplate reader after 10 min. The H_2O_2 release was determined by comparison with a calibration curve using known amounts of H_2O_2 (0–25 µM); H_2O_2 concentration was determined using $\varepsilon_{240\ nm} = 39.4\ M^{-1}.cm^{-1}$ [58].

Superoxide anion formed by nNOS was detected by spin-trapping with the cyclic nitrone DEPMPO and EPR detection of the spin adducts. A typical incubation mixture (final volume, 100 µL) contained 1 mM NADPH, 1.0 mM $CaCl_2$, 10 µg/mL CaM, and 50 mM DEPMPO in 50 mM Hepes buffer pH 7.4. Five-10 µL of nNOS (final concentration, 100-200 nM) were mixed with the previous mixture that was rapidly transferred into an Aqua-X sample cell fitted in a shq001 cavity of a X-band Bruker EPR Elexsys 500 spectrometer (Bruker, Wissembourg, France) maintained at 21°C and using the following instrument settings: field modulation frequency, 100 kHz; field modulation amplitude, 0.2 mT; time constant, 40.96 ms; microwave power, 10 mW; field width, 140 mT; center field, 349 T; scan time, 41.94 s; number of scans, 16. DEPMPO-OOH spectrum was identified by comparison with incubations performed in the presence of xanthine/ xanthine oxidase. The changes in amplitude of the first peak of the DEPMPO-OOH adduct were used to quantify the amounts of superoxide generated in the experiments.

Cell culture and treatments

Endothelial (HUVECs) were cultured in P100 dishes or 96-well plates (10^4cells/well) at 37 °C to ~95% confluence in Eagle's Minimal Essential Medium containing 10% fetal calf serum (FCS), 5 mM glutamine, penicillin–streptomycin (100 U/mL), 2.5 µg/ mL amphotericin, and 125 µg/mL gentamycin. Human melanocyte suspensions were obtained from foreskins of Caucasian children as previously described [59]. Human primary melanocytes were grown in MCDB153 medium supplemented with 2% FCS, 0.5 mg/ml hydrocortisone, 5 mg/ml insulin, 16 nM phorbol-12 myristate 13-acetate, 1 ng/ml basic fibroblast growth factor, 20 mg/ml bovine pituitary extract, 10 mM forskolin, and penicillin/ streptomycin (100U/ml /50 mg/ml) [60]. Human A375 (CRL-1619) melanoma cells were purchased from American Tissue Culture Collection (Molsheim, France) and grown in RPMI medium supplemented with 10% FCS and penicillin/streptomycin (100U / ml /50 mg/ml). For each experiment, cells were starved without 1% FCS in appropriate medium during 14 h before drug (NS1) stimulation.

Preparation of mouse isolated aorta

Aorta rings were dissected from 15 weeks-old male C57Bl/6J mice (Elevage Janvier, Le Genest-St-Isle, France) anesthetized with ketamine/ xylazine before sacrifice. All experimental procedures and protocols were approved by the local Ethics Committee according to National Care Regulations.

Cell viability tests

Serial dilutions of NS1 (1µM – 100 µM) were added to the HUVEC that were further incubated at 37°C for up to 24 or 48 hours. The cell supernatants were removed and replaced by fresh medium before MTT analysis. At the chosen time, 20 µL of 5 mg/mL MTT in PBS was added to the cells and further incubated at 37 °C. After washing, 100 µL of DMSO were added in each well and absorbance at 570 nm was measured on a multi-well Plate Reader (Model VICTOR™ X4, PerkinElmer) with subtraction of blank value at 630 nm. In additional experiments, HUVECs growth cultured in the presence or absence of NS1 was recorded by videomicroscopy using an Axiocam camera.

Cell viability of MHN and melanoma cells

For the study of the differential effect of NS1 on melanocytes and melanoma A375 cells, viable cells were counted using trypan blue dye exclusion method as previously described [54].

Measurements of nitric oxide production from isolated aortic rings in situ by spin trapping and Electron Paramagnetic Resonance (EPR) spectroscopy

Production of NO was assayed by spin trapping in presence of the spin trap ([Fe^{II}-$(DETC)_2$] and EPR detection of the $Fe^{II}(DETC)_2NO$ paramagnetic NO adduct by EPR spectroscopy. Isolated aortic rings were incubated in KREBS-Hepes buffer in the presence of NS1 or vehicle alone (1h at 37°C) and stimulated or not during 30 minutes with calcium ionophore (A23187, 2 µM or ionomycin, 2 µM) at 37°C [61]. The EPR signal of the [$Fe^{II}NO$-$(DETC)_2$] complex (g_1 = 2.035; A_{hfs} = 1.3 mT) was recorded on a Bruker EMX100 spectrometer spectrometer (X-band, microwave frequency ~9.35 GHz, modulation frequency, 100 kHz) with following settings: microwave power (MP), 20 mW; modulation amplitude (MA), 0.5 mT; time constant (TC) 163 ms; 10 scans, 77°K.

Measurements of superoxide anion production from isolated aortic rings by EPR spectroscopy

Superoxide anion formation was assayed by an EPR method using the spin probe CAT1-H being a cell non-permeable spin probe [62] with some modification: aortic rings (about 1-mm length) were dissected from mouse thoracic artery, preincubated on ice (1 hour) in KREBS-DTPA-Hepes buffer (0.1 mM DTPA, 20 mM HEPES, pH 7.5) with or without NS1, catalase (100U / ml) or SOD (100U/mL), and inserted into the capillary after addition of 5 mM CAT1-H. The kinetics of EPR signals formation of the CAT1● species were recorded on-line during 10 minutes at 37°C in capillary interposed into the cavity of the EPR spectrometer (MiniScop MS400, Magnetech, equipped with bio-temperature controller). The following instrumental settings were used, modulation frequency, 100 kHz; microwave frequency, ~9.4 GHz; MP, 20 mW; MA, 0.1 mT. The rates of aortic basal ROS formation were calculated as a slope of linearized kinetic curve, obtained from automatically recording of radical signal formation (first hf component) using Magnettech kinetic software, and normalized by the length of aortic rings or protein content after subtraction of the basal signal (without aortic rings). Preincubation of aortic rings with SOD (100U/ml, 30 minutes on ice) decreased EPR signal accumulation by 80%. CAT1-H reaction with DPI ruled out the use of DPI to test the role of NOX in NS1-induced decrease of superoxide ion.

Fluorescence detection of ROS generation

CellROX® Deep Red Reagent (Invitrogen) was used for oxidative stress detection (absorption and emission maxima at ~644/665 nm). HUVECs (1-1.5x10^5 cells) plated in 12-well plates at ~85% confluence were pre-incubated with increasing concentrations of NS1 for 30 min at 37°C, and then further incubated with 2 µM CellROX® Deep Red Reagent for 30 min. Tert-butyl hydroperoxide (TBHP) (Sigma), an oxidative stress inducer, was used as a positive control. After centrifugation at 1,000 rpm for 5 min, the cell pellets were resuspended in PBS containing 30% of enzyme-free Cell Dissociation Buffer (Gibco®) and analysed by FACS Calibur flow cytometer (Becton-Dickinson). To address the modulation effect of NS1 on ROS formation, the MFI (Mean Fluorescence Intensity) related to fluorescence emission of the CellROX® Deep Red Reagent, detected in FL-4 channel and measured in the presence of a given concentration of NS1, was normalized by the MFI obtained in the absence of NS1 (*i.e.* CellROX® Deep Red Reagent alone; also measured in FL-4 channel), giving the fluorescence enhancement factor. Similar measurements were performed in A375 melanoma cells.

Angiogenesis assays

To assess *in vitro* the angiogenic process, an assay of endothelial network formation by plating of endothelial cells (HUVECs (passage 3-5)) on Matrigel was used as previously reported [19]**.** Results were expressed as endothelial network percentage ± SEM versus control condition and *n* reflects the number of experiments.

Western blots, NO measurements and cell cycle study in A375 and MHN

Cell cycle profiles were determined by flow cytometric analysis of DAPI-stained cells as described before [44]. Detection of NOx (NO and NO-derived species as nitrite and nitrate) in the supernatants of melanoma and melanocytes were detected by the Griess method using the manufacturer protocol. Cell cycle profiles were collected using a FACScan instrument and analyzed with the CELLQUEST software (Becton-Dickinson, Le Pont de Claix, France). Western blot analyses were performed as described [54].

Statistical analysis

All data were presented as means ± standard deviation (SD). Statistical analysis was performed using the Student t-test: * $P < 0.05$ versus control, ** $P< 0.01$ vs control, *** $P < 0.001$ deemed statistically significant.

Source of support

This work was supported by Agence Nationale de la Recherche (ANR-PCVI08-006-01, TRIGNOSTUMOR) to AS (PI), JLB and ED.

Financial Disclosure, Disclosure

There is no conflict of interest.

Abbreviations

CaM, calmodulin; CAT-1H, 1-hydroxy-2,2,6,6-tetramethylpiperidin-4-yl-trimethylammonium chloride; DEPMPO, 5-diethoxyphosphoryl-5-methyl-1-pyrroline N-oxide; Na-DETC, diethyldithiocarbamate, sodium salt; DTPA, diethylenetriaminepentaacetic acid; EPR, electron paramagnetic resonance; FCS, fetal calf serum; HUVEC, human umbilical vein endothelial cells; n-, i-, and eNOS, neuronal, inducible and endothelial nitric oxide synthase, respectively; H4B, (6R)-5,6,7,8-tetrahydrobiopterin; L-NAME, L-NG-nitroarginine methyl ester; MTT, 3-[4,5-dimethylthiazol-2-yl]-2,5-diphenyl tetrazolium bromide; NHM, normal human melanocytes; NOX, NADPH oxidase; ROS, reactive oxygen species; TBHP, tert-butyl hydroxyperoxide.

REFERENCES

1. Atochin DN and Huang PL. Endothelial nitric oxide synthase transgenic models of endothelial dysfunction. Pflugers Arch. 2010; 460(6):965-974.
2. Frey RS, Ushio-Fukai M and Malik AB. NADPH oxidase-dependent signaling in endothelial cells: role in physiology and pathophysiology. Antioxid Redox Signal. 2009; 11(4):791-810.
3. Kao CL, Chen LK, Chang YL, Yung MC, Hsu CC, Chen YC, Lo WL, Chen SJ, Ku HH and Hwang SJ. Resveratrol protects human endothelium from H(2)O(2)-induced oxidative stress and senescence via SirT1 activation. J Atheroscler Thromb. 2010; 17(9):970-979.
4. Ischiropoulos HB, JS. Oxidative stress and nitration in neurodegeneration: cause, effect, or association? J Clin Invest. 2003; 111(2):163-169.
5. Moncada S, Palmer, R.M., Higgs, E.A. Nitric oxide: physiology, pathophysiology and pharmacology. Pharmacol Rev. 1991; 43(2):109-142.
6. Li H and Forstermann U. Prevention of atherosclerosis by interference with the vascular nitric oxide system. Curr Pharm Des. 2009; 15(27):3133-3145.
7. Kostourou V, Cartwright JE, Johnstone AP, Boult JK, Cullis ER, Whitley G and Robinson SP. The role of tumour-derived iNOS in tumour progression and angiogenesis. Br J Cancer. 2011; 104(1):83-90.
8. Patenaude A, Fuller M, Chang L, Wong F, Paliouras G, Shaw R, Kyle AH, Umlandt P, Baker JH, Diaz E, Tong J, Minchinton AI and Karsan A. Endothelial-specific Notch blockade inhibits vascular function and tumor growth through an eNOS-dependent mechanism. Cancer Res. 2014; 74(9):2402-2411.
9. Wind S, Beuerlein K, Eucker T, Muller H, Scheurer P, Armitage ME, Ho H, Schmidt HH and Wingler K. Comparative pharmacology of chemically distinct NADPH oxidase inhibitors. Br J Pharmacol. 2010; 161(4):885-898.
10. Poulos TL and Li H. Structural basis for isoform-selective inhibition in nitric oxide synthase. Acc Chem Res. 2013; 46(2):390-398.
11. Li Y, Wang H, Tarus B, Perez MR, Morellato L, Henry E, Berka V, Tsai AL, Ramassamy B, Dhimane H, Dessy C, Tauc P, Boucher JL, Deprez E and Slama-Schwok A. Rational design of a fluorescent NADPH derivative imaging constitutive nitric-oxide synthases upon two-photon excitation. Proc Natl Acad Sci U S A. 2012; 109(31):12526-12531.
12. Beaumont E, Lambry JC, Blanchard-Desce M, Martasek P, Panda SP, van Faassen EE, Brochon JC, Deprez E and Slama-Schwok A. NO formation by neuronal NO-synthase

can be controlled by ultrafast electron injection from a nanotrigger. Chembiochem. 2009; 10(4):690-701.

13. Beaumont E, Lambry JC, Gautier C, Robin AC, Gmouh S, Berka V, Tsai AL, Blanchard-Desce M and Slama-Schwok A. Synchronous photoinitiation of endothelial NO synthase activity by a nanotrigger targeted at its NADPH site. J Am Chem Soc. 2007; 129(7):2178-2186.
14. Beaumont E, Lambry JC, Robin AC, Martasek P, Blanchard-Desce M and Slama-Schwok A. Two photon-induced electron injection from a nanotrigger in native endothelial NO-synthase. Chemphyschem. 2008; 9(16):2325-2331.
15. Lambry JC, Beaumont E, Tarus B, Blanchard-Desce M and Slama-Schwok A. Selective probing of a NADPH site controlled light-induced enzymatic catalysis. J Mol Recognit. 2010; 23(4):379-388.
16. Vanin AF. Iron diethyldithiocarbamate as spin trap for nitric oxide detection. Methods Enzymol. 1999; 301:269-279.
17. Pfeiffer S, Leopold E, Schmidt K, Brunner F and Mayer B. Inhibition of nitric oxide synthesis by NG-nitro-L-arginine methyl ester (L-NAME): requirement for bioactivation to the free acid, NG-nitro-L-arginine. Br J Pharmacol. 1996; 118(6):1433-1440.
18. Tuynman A, Perollier C, Frapart Y, Schumann-Bard P, Collot V, Rault S and Boucher JL. Inhibitory effects and spectral interactions of isomeric methoxyindazoles on recombinant nitric oxide synthases. Nitric Oxide. 2003; 9(2):86-94.
19. Brouet A, Sonveaux P, Dessy C, Moniotte S, Balligand JL and Feron O. Hsp90 and caveolin are key targets for the proangiogenic nitric oxide-mediated effects of statins. Circ Res. 2001; 89(10):866-873.
20. Dessy C, Saliez J, Ghisdal P, Daneau G, Lobysheva, II, Frerart F, Belge C, Jnaoui K, Noirhomme P, Feron O and Balligand JL. Endothelial beta3-adrenoreceptors mediate nitric oxide-dependent vasorelaxation of coronary microvessels in response to the third-generation beta-blocker nebivolol. Circulation. 2005; 112(8):1198-1205.
21. Stuehr DJ, Wei CC, Santolini J, Wang Z, Aoyagi M and Getzoff ED. Radical reactions of nitric oxide synthases. Biochem Soc Symp. 2004; (71):39-49.
22. Liu-Smith F, Dellinger, R.,and Meyskens, F.L., Jr. Updates of reactive oxygen species in melanoma etiology and progression. Arch Biochem Biophys. 2014; in press.
23. Drummond GR, Selemidis S, Griendling KK and Sobey CG. Combating oxidative stress in vascular disease: NADPH oxidases as therapeutic targets. Nat Rev Drug Discov. 2011; 10(6):453-471.
24. Craige SM, Chen, K., Pei, Y., Huang, X., Chen, C., Shibata, R., Sato, K., Walsh, K., Keaney, J.F., Jr. NADPH oxidase 4 promotes endothelial angiogenesis though endothelial nitric oxide synthase activation. Circulation. 2011; 124:731-740.
25. Godoy LC, Anderson CT, Chowdhury R, Trudel LJ and Wogan GN. Endogenously produced nitric oxide mitigates sensitivity of melanoma cells to cisplatin. Proc Natl Acad Sci U S A. 2012; 109(50):20373-20378.
26. Melo FH, Molognoni F, Morais AS, Toricelli M, Mouro MG, Higa EM, Lopes JD and Jasiulionis MG. Endothelial nitric oxide synthase uncoupling as a key mediator of melanocyte malignant transformation associated with sustained stress conditions. Free Radic Biol Med. 2011; 50(10):1263-1273.
27. Yamaura M, Mitsushita J, Furuta S, Kiniwa Y, Ashida A, Goto Y, Shang WH, Kubodera M, Kato M, Takata M, Saida T and Kamata T. NADPH oxidase 4 contributes to transformation phenotype of melanoma cells by regulating G2-M cell cycle progression. Cancer Res. 2009; 69(6):2647-2654.
28. Burch PM and Heintz NH. Redox regulation of cell-cycle re-entry: cyclin D1 as a primary target for the mitogenic effects of reactive oxygen and nitrogen species. Antioxid Redox Signal. 2005; 7(5-6):741-751.
29. Napoli C, Paolisso G, Casamassimi A, Al-Omran M, Barbieri M, Sommese L, Infante T and Ignarro LJ. Effects of nitric oxide on cell proliferation: novel insights. J Am Coll Cardiol. 2013; 62(2):89-95.
30. Garcin ED, Bruns CM, Lloyd SJ, Hosfield DJ, Tiso M, Gachhui R, Stuehr DJ, Tainer JA and Getzoff ED. Structural basis for isozyme-specific regulation of electron transfer in nitric-oxide synthase. J Biol Chem. 2004; 279(36):37918-37927.
31. Sonveaux P, Martinive P, DeWever J, Batova Z, Daneau G, Pelat M, Ghisdal P, Gregoire V, Dessy C, Balligand JL and Feron O. Caveolin-1 expression is critical for vascular endothelial growth factor-induced ischemic hindlimb collateralization and nitric oxide-mediated angiogenesis. Circ Res. 2004; 95(2):154-161.
32. Lim KH, Ancrile BB, Kashatus DF and Counter CM. Tumour maintenance is mediated by eNOS. Nature. 2008; 452(7187):646-649.
33. Meierjohann S. Oxidative stress in melanocyte senescence and melanoma transformation. Eur J Cell Biol. 2013.
34. Govindarajan B, Sligh JE, Vincent BJ, Li M, Canter JA, Nickoloff BJ, Rodenburg RJ, Smeitink JA, Oberley L, Zhang Y, Slingerland J, Arnold RS, Lambeth JD, Cohen C, Hilenski L, Griendling K, et al. Overexpression of Akt converts radial growth melanoma to vertical growth melanoma. J Clin Invest. 2007; 117(3):719-729.
35. Liu F, Gomez Garcia AM and Meyskens FL, Jr. NADPH oxidase 1 overexpression enhances invasion via matrix metalloproteinase-2 and epithelial-mesenchymal transition in melanoma cells. J Invest Dermatol. 2012; 132(8):2033-2041.
36. Florio T, Morini M, Villa V, Arena S, Corsaro A, Thellung S, Culler MD, Pfeffer U, Noonan DM, Schettini G and Albini A. Somatostatin inhibits tumor angiogenesis and growth via somatostatin receptor-3-mediated regulation of endothelial nitric oxide synthase and mitogen-

activated protein kinase activities. Endocrinology. 2003; 144(4):1574-1584.

37. Ying L and Hofseth LJ. An emerging role for endothelial nitric oxide synthase in chronic inflammation and cancer. Cancer Res. 2007; 67(4):1407-1410.

38. Lahdenranta J, Hagendoorn J, Padera TP, Hoshida T, Nelson G, Kashiwagi S, Jain RK and Fukumura D. Endothelial nitric oxide synthase mediates lymphangiogenesis and lymphatic metastasis. Cancer Res. 2009; 69(7):2801-2808.

39. Feng J, Damrauer SM, Lee M, Sellke FW, Ferran C and Abid MR. Endothelium-dependent coronary vasodilatation requires NADPH oxidase-derived reactive oxygen species. Arterioscler Thromb Vasc Biol. 2010; 30(9):1703-1710.

40. Mochizuki T, Furuta S, Mitsushita J, Shang WH, Ito M, Yokoo Y, Yamaura M, Ishizone S, Nakayama J, Konagai A, Hirose K, Kiyosawa K and Kamata T. Inhibition of NADPH oxidase 4 activates apoptosis via the AKT/ apoptosis signal-regulating kinase 1 pathway in pancreatic cancer PANC-1 cells. Oncogene. 2006; 25(26):3699-3707.

41. Salmeen A, Park BO and Meyer T. The NADPH oxidases NOX4 and DUOX2 regulate cell cycle entry via a p53-dependent pathway. Oncogene. 2010; 29(31):4473-4484.

42. Luo L, Kaur Kumar J and Clement MV. Redox control of cytosolic Akt phosphorylation in PTEN null cells. Free Radic Biol Med. 2012; 53(9):1697-1707.

43. Zhang C, Lan, T. Hou, J., Li, J., Fang, R., Yang, Z., Zhang, J., Liu, B. NOX4 promotes non-small cell lung cancer cell proliferation and metastasis through positive feedback regulation of PI3K/ Akt signalling. Oncotarget. 2014; 5(12):4392-4405.

44. Fenouille N, Puissant A, Tichet M, Zimniak G, Abbe P, Mallavialle A, Rocchi S, Ortonne JP, Deckert M, Ballotti R and Tartare-Deckert S. SPARC functions as an anti-stress factor by inactivating p53 through Akt-mediated MDM2 phosphorylation to promote melanoma cell survival. Oncogene. 2011; 30(49):4887-4900.

45. Liu P, Begley M, Michowski W, Inuzuka H, Ginzberg M, Gao D, Tsou P, Gan W, Papa A, Kim BM, Wan L, Singh A, Zhai B, Yuan M, Wang Z, Gygi SP, et al. Cell-cycle-regulated activation of Akt kinase by phosphorylation at its carboxyl terminus. Nature. 2014; 508(7497):541-545.

46. Mounir Z, Krishnamoorthy JL, Wang S, Papadopoulou B, Campbell S, Muller WJ, Hatzoglou M and Koromilas AE. Akt determines cell fate through inhibition of the PERK-eIF2alpha phosphorylation pathway. Sci Signal. 2011; 4(192):ra62.

47. Piccoli C, D'Aprile A, Ripoli M, Scrima R, Lecce L, Boffoli D, Tabilio A and Capitanio N. Bone-marrow derived hematopoietic stem/progenitor cells express multiple isoforms of NADPH oxidase and produce constitutively reactive oxygen species. Biochem Biophys Res Commun. 2007; 353(4):965-972.

48. Csanyi G, Taylor WR and Pagano PJ. NOX and inflammation in the vascular adventitia. Free Radic Biol Med. 2009; 47(9):1254-1266.

49. Garrido-Urbani S, Jemelin S, Deffert C, Carnesecchi S, Basset O, Szyndralewiez C, Heitz F, Page P, Montet X, Michalik L, Arbiser J, Ruegg C, Krause KH and Imhof BA. Targeting vascular NADPH oxidase 1 blocks tumor angiogenesis through a PPARalpha mediated mechanism. PLoS One. 2011; 6(2):e14665.

50. Altenhofer S, Kleikers PW, Radermacher KA, Scheurer P, Rob Hermans JJ, Schiffers P, Ho H, Wingler K and Schmidt HH. The NOX toolbox: validating the role of NADPH oxidases in physiology and disease. Cell Mol Life Sci. 2012; 69(14):2327-2343.

51. Silverman RB. Design of selective neuronal nitric oxide synthase inhibitors for the prevention and treatment of neurodegenerative diseases. Acc Chem Res. 2009; 42(3):439-451.

52. Huang H, Li H, Yang S, Chreifi G, Martasek P, Roman LJ, Meyskens FL, Poulos TL and Silverman RB. Potent and selective double-headed thiophene-2-carboximidamide inhibitors of neuronal nitric oxide synthase for the treatment of melanoma. J Med Chem. 2014; 57(3):686-700.

53. Jain RK. Normalizing Tumor Microenvironment to Treat Cancer: Bench to Bedside to Biomarkers. J Clin Oncol. 2013; 31(17):2205-18

54. Tomic T, Botton T, Cerezo M, Robert G, Luciano F, Puissant A, Gounon P, Allegra M, Bertolotto C, Bereder JM, Tartare-Deckert S, Bahadoran P, Auberger P, Ballotti R and Rocchi S. Metformin inhibits melanoma development through autophagy and apoptosis mechanisms. Cell Death Dis. 2011; 2:e199.

55. Slama-Schwok A, Boucher JL, Xu-Li Y and Deprez E. (2014). New family of analogues and nadp+ or nadph, their preparation and their application in therapeutics. US Patent US20140193339 A1 and PCT/EP2012/061744).

56. Moali C, Boucher JL, Sari MA, Stuehr DJ and Mansuy D. Substrate specificity of NO synthases: detailed comparison of L-arginine, homo-L-arginine, their N omega-hydroxy derivatives, and N omega-hydroxynor-L-arginine. Biochemistry. 1998; 37(29):10453-10460.

57. Heinzel B, John M, Klatt P, Bohme E and Mayer B. Ca2+/ calmodulin-dependent formation of hydrogen peroxide by brain nitric oxide synthase. Biochem J. 1992; 281 (Pt 3):627-630.

58. Nelson DP and Kiesow LA. Enthalpy of decomposition of hydrogen peroxide by catalase at 25 degrees C (with molar extinction coefficients of H_2O_2 solutions in the UV). Anal Biochem. 1972; 49(2):474-478.

59. Botton T, Puissant A, Bahadoran P, Annicotte JS, Fajas L, Ortonne JP, Gozzerino G, Zamoum T, Tartare-Deckert S, Bertolotto C, Ballotti R and Rocchi S. *In vitro* and *in vivo* anti-melanoma effects of ciglitazone. J Invest Dermatol. 2009; 129(5):1208-1218.

60. Botton T, Puissant A, Cheli Y, Tomic T, Giuliano S, Fajas L, Deckert M, Ortonne JP, Bertolotto C, Tartare-Deckert S,

Ballotti R and Rocchi S. Ciglitazone negatively regulates CXCL1 signaling through MITF to suppress melanoma growth. Cell Death Differ. 2011; 18(1):109-121.

61. Lobysheva I, Rath G, Sekkali B, Bouzin C, Feron O, Gallez B, Dessy C and Balligand JL. Moderate caveolin-1 downregulation prevents NADPH oxidase-dependent endothelial nitric oxide synthase uncoupling by angiotensin II in endothelial cells. Arterioscler Thromb Vasc Biol. 2011; 31(9):2098-2105.
62. Dikalov S, Griendling KK and Harrison DG. Measurement of reactive oxygen species in cardiovascular studies. Hypertension. 2007; 49(4):717-727.
